# Performance of a Native Butterfly and Introduced Moth on Native and Introduced Lineages of *Phragmites australis*

**DOI:** 10.3390/insects12121102

**Published:** 2021-12-09

**Authors:** Adam M. Lambert, Lisa A. Tewksbury, Richard A. Casagrande

**Affiliations:** 1 Marine Science Institute, University of California, Santa Barbara, CA 93106, USA; 2 Department of Plant Sciences and Entomology, University of Rhode Island, Kingston, RI 02881, USA; lisat@uri.edu (L.A.T.); casa@uri.edu (R.A.C.)

**Keywords:** artificial diet, biological control, common reed, haplotype, herbivore preference, invasive species, survivorship curve

## Abstract

**Simple Summary:**

We evaluated the survival and development of a native butterfly (*Poanes viator*) and introduced moth (*Rhizedra lutosa*) fed native or introduced lineages of common reed (*Phragmites australis*). The native butterfly feeds more generally on monocots, so some larvae were also fed wild rice (*Zizania aquatica).* Using *R. lutosa*, we also tested an artificial diet supplemented with common reed rhizome powder as a potential food for rearing stalk and rhizome boring Lepidoptera. Generally, both insect species had low larval survival on all plants used, but high pupation success once insects reached that stage. Only *P. viator* larvae reared on leaves from native plants pupated and completed development. *Rhizedra lutosa* completed all developmental stages on native and introduced *P. australis* rhizomes. The artificial diet resulted in a doubling of survival and successful development to adults for *R. lutosa*. Several specialist Lepidopteran species are being considered for approval as biological control agents for the non-native, invasive common reed in North America, and the convenience and increased larval performance make this artificial diet a good alternative for rearing organisms.

**Abstract:**

This study examined the performance of *Poanes viator* (Edwards) (Hesperiidae), a native North American skipper, and *Rhizedra lutosa* (Hübner) (Noctuidae), an introduced moth, reared on native and non-native, invasive lineages of *Phragmites australis*. *Poanes viator* is a generalist on monocots and larvae were also fed leaves of *Zizania aquatica*, a native macrophyte that the skipper commonly uses as a host plant. Larval survival and duration, pupal weight, and pupation time were compared for *P. viator* feeding on leaf tissue and *R. lutosa* feeding on rhizomes of either native or introduced plants. We also tested an artificial diet supplemented with *P. australis* rhizome powder as a potential food for rearing other stalk and rhizome boring Lepidoptera. In experiments using excised plant tissues, some individuals of both species fed and developed to the pupal stage on native and introduced plants, but overall, larval survival rates were low. Plant species/haplotype identity did not cause strong differences in larval survival for either species. However, *P. viator* larvae only pupated when feeding on native plants (*Zizania aquatica* and native *P. australis* haplotypes), whereas *R. lutosa* successfully pupated on both native and introduced *P. australis*. Although larval survival was low, 100% of *P. viator* and 95% of *R. lutosa* that reached the pupal stage emerged as adults. *Rhizedra lutosa* larvae fed an artificial diet supplemented with *P. australis* rhizome powder had significantly greater survival and pupal weights, and shorter pupation times than larvae fed rhizomes only. Several specialist Lepidopteran species are being considered for approval as biological control agents for the non-native *P. australis* haplotype, and the convenience and increased larval performance make this artificial diet a good alternative for rearing organisms.

## 1. Introduction

*Phragmites australis* (Cav.) (common reed) is a large wetland grass with a worldwide distribution. A European *P. australis* haplotype was introduced to the east coast of the United States in the late 1800s and continues to spread aggressively in freshwater and salt marsh ecosystems across North America [1,2]. The native lineage (*P. australis* subsp. *americanus*) consists of numerous haplotypes with a high degree of geographic structuring, and population declines in eastern North America are associated with the rapid expansion of the European haplotype [2]. The adverse ecological effects of introduced *P. australis* invasion (reviewed in [3,4] have heightened interest in control of this plant.

Efforts are underway to develop biological control using European insects to target the introduced lineage, both to reduce its impacts to wetland ecosystems and protect the sympatric native subspecies [5,6,7]. Host specificity at the genetic level (haplotype specificity) will be critical to the success of this program [5,8,9]. Over 30 insect species are documented to feed to varying degrees on native and introduced *P. australis* lineages [7], and there is emerging evidence that differences in the nutritional quality and defensive traits of native and introduced haplotypes can drive the structuring of these insect communities [10,11]. In addition to ensuring haplotype (or lineage) specificity when developing a new biocontrol agent, we would want to ensure agents will perform well with high survival on the intended targets. Whether haplotypes impact herbivore survival has yet to be empirically tested in this system and we aimed to investigate this here by evaluating a native North American skipper and an introduced noctuid moth.

The broad-winged skipper, *Poanes viator* (Edwards) (Hesperiidae), is a marsh-dwelling butterfly native to eastern North America. Larvae of these butterflies feed on the leaves of wetland plants including *P. australis*, wild rice (*Zizania aquatica*), sedges (*Carex* spp.), and marsh millet (*Zizaniopsis miliacea*) [12,13]. Shapiro [13] identified two distinct subspecies; *P. v. viator*, an inland species with a distribution throughout the northeast and midwest USA, and *P. v. zizaniae*, a coastal plain species distributed along the Atlantic coast and along the Gulf Coast to Texas. This species has recently increased its range by including *P. australis* (likely the introduced lineage) in its diet [14] and larvae and adults are common in stands of introduced *P. australis* throughout coastal southern New England (A. Lambert, personal observation). We previously observed adults in native *P. australis* stands at our study sites on Block Island, Rhode Island in 2004; however, larval feeding has not been confirmed at these sites.

*Rhizedra lutosa* (Hübner) (Noctuidae) is a specialist herbivore on *P. australis* that is native to Europe and an accidental introduction into North America from unknown sources. Larvae feed on newly emerged shoots before descending into and feeding on the rhizomes [15] where they consume all but the thin outer rhizome epidermis, killing all attached shoots. In the Netherlands, *R. lutosa* causes severe damage toward the upland side of *P. australis* stands where soils are not saturated [15]. Moths were first reported in New Jersey in 1988 [16] and have subsequently been found throughout the northeast US [17,18]. Population densities have remained relatively constant following introduction, having minimal impact on *P. australis* stands [17]. Previously, there was no evidence that *R. lutosa* feeds on native *P. australis* haplotypes.

To elucidate the effects of plant lineage on insect performance, we tested two hypotheses: (1) performance of the native generalist *P. viator* is similar on native and introduced *P. australis* due to its ability to incorporate multiple monocot plant species in its diet; and (2) the introduced specialist *R. lutosa* will have increased performance on introduced *P. australis* due to its evolutionary history of using this haplotype in its native range. We also reared *R. lutosa* larvae on an artificial diet containing either native or introduced *P. australis* to determine if the diet is suitable for rearing this species and other Lepidoptera that feed in *P. australis* rhizomes and stems.

## 2. Materials and Methods

### 2.1. Plant Collection and Propagation

*Phragmites australis* rhizomes were collected from Montezuma National Wildlife Refuge, New York (introduced haplotype M and native haplotype E), Holt Research Forest, Arrowsic, Maine (native haplotype E), Galilee, Rhode Island (introduced haplotype M) in the late winter of 2002, and from Block Island, Rhode Island (native haplotype AB) in September 2002. The haplotypes of these populations were previously confirmed as part of the study by Saltonstall [2]. Three rhizome fragments with four to five nodes each were planted in a 50/50 (volume) sand/peat mix in a 27 cm in diameter × 44 cm in height pots. Pots were placed in 1.5 m in diameter × 0.15 m in depth plastic pools filled with water and maintained outdoors at a common garden facility at the University of Rhode Island. Water level was routinely added to all pools to maintain continuous saturation in all pots. One gram of Osmocote^®^ timed release fertilizer (14N:14P:14K) per plant was used as a top dressing and plants were watered daily until the soil was saturated. Wild rice (*Zizania aquatica* L.; native to North America) seeds were collected in late August 2003 from populations in Uxbridge, MA, and vernalized in water for 7 months at 4 °C. Sprouting seeds were planted in a 50/50 (volume) sand/peat mix in 5 L nursery pots and grown in water-filled pools (as above). 

### 2.2. Insect Collection and Rearing

*Poanes viator* adults were collected in July and August 2003 from *P. australis* (haplotype M) stands in Coventry and Narragansett, Rhode Island. Adults were sorted by sex, and half of the females were added to each of two 61 cm × 61 cm × 61 cm screen cages in the laboratory for oviposition. Insects were kept at ambient temperature (approximately 25 °C) with a 16:8 L:D photoperiod provided by fluorescent lights. Moistened paper towels were placed on the bottom of each cage, and cages were misted with water daily to increase humidity above ambient conditions. *Clethra alnifolia* and *Buddleja davidii* flowers were provided as a nectar source using cut stems placed in water. Ten to fifteen *P. australis* (haplotype M) stems with leaves were provided in each cage for oviposition. Only the top portion of stems with new growth was used, and stems and leaves were rinsed with deionized water and dried with paper towel before adding to cages. Eggs were collected daily by excising leaf sections containing the eggs and kept in covered 44 mL plastic cups with moistened filter paper at ambient temperature until hatching. Each newly hatched larva was placed on a piece of filter paper in a covered 237 mL plastic cup and randomly assigned to a leaf-feeding treatment consisting of one of the *P. australis* haplotypes described above or *Z. aquatica*. Leaves from the plants grown at the common garden facility were used for the majority of the experiment except in the fall when plants senesced, when leaves were taken from greenhouse plants. Larvae were fed one leaf excised from the stem at a time and leaves were checked daily for larval feeding and replaced as necessary. No additional moisture source was used, as the covered cups reduced evaporative loss and helped maintain leaf turgor. Insects were maintained in a growth chamber at 23 °C day:15 °C night on a 16:8 L:D photoperiod. Larval survival, pupation time, and pupation weight were recorded.

*Rhizedra lutosa* adults were collected in September through November 2003 from salt marshes in Narragansett and Charlestown, Rhode Island using black light traps. Females were added to each of two oviposition cages and maintained under ambient laboratory conditions. A 10% honey solution was provided in each cage. *Phragmites australis* (haplotype M) stems with leaves were provided for oviposition and cages were misted with water daily to increase humidity. Eggs were collected daily by excising leaves around egg masses and placed in covered 44 mL plastic cups with moistened filter paper. Eggs were maintained in covered cups in a refrigerator at 4 °C for five to seven months. In April 2004, eggs were transferred to 23 °C until hatching. Newly hatched larvae were randomly assigned to *P. australis* rhizome feeding treatments using the same haplotypes listed above. The largest rhizomes available in each pot were washed and sectioned between nodes leaving one internode section intact. Rhizomes were placed in 237 mL plastic cups with a piece of filter paper, covered, and one newly hatched larva was placed inside the rhizome fragment with a paintbrush. Block Island plants (native haplotype AB) did not grow well throughout the experimental period; so field-collected rhizomes were used periodically in Block Island feeding treatments following the same methods as above. Near the end of the experiment, larvae were too large to fit in native New York and Maine rhizomes, and rhizomes had to be split in half before feeding caterpillars. Insects were maintained in a growth chamber at 23 °C:5 °C day: night cycle on a 16:8 L:D photoperiod. Larvae in rhizomes were checked daily and rhizomes were changed every five days or earlier when all inner rhizome surfaces were eaten. Larval survival and pupation time and weight were measured, as well as fresh weight of larvae at 35 days and 70 days.

Newly hatched *R. lutosa* larvae were also reared on artificial diets containing *P. australis* haplotypes. Artificial diet # F9768 was obtained from BioServ Inc. (Frenchtown, NJ, USA). This diet, formulated for corn stalk borers, contains corn stalk powder, agar, casein, soy flour, Brewer’s yeast, Wesson salt mix, sucrose, sorbic acid, wheat germ, cellulose, vitamin mix, ascorbic acid, methylparaben, and KOH solution. In place of the corn stalk powder, rhizome powder from introduced (haplotype M) or native *P. australis* (haplotypes E or AB) was added to the diet at a rate of 14.2 g/L. To obtain rhizome powder, the outer rhizome layer was removed using a potato peeler and rhizomes were dried at 60 °C to constant weight. Rhizomes were then ground to pass through a 20-mesh screen in a Wiley mill. The diet was dispensed into 30 mL plastic cups with lids and stored at 4 °C. For experimentation, cups were removed from storage and allowed to warm to ambient laboratory temperature before a single larva was added to each cup using a randomized design to assign larvae to each of the three diets. The experiment was conducted in a growth chamber at 23 °C:5 °C day: night cycle on a 16:8 L:D photoperiod. Cups were checked daily and larvae were transferred to cups with new diet when 75% of the diet was eaten or if there was evidence of bacterial or fungal growth. Independent variables were measured as above.

### 2.3. Field Surveys for Rhizedra lutosa

To determine if *R. lutosa* larvae were present in native *P. australis* stands, we sampled two native and two introduced stands on Block Island, Rhode Island in May 2004. We also sampled these four stands, and one native stand and one introduced stand in Wells, Maine from June to September in 2005. *Rhizedra lutosa* damage can be assessed in early spring as bore holes on newly emerged shoots and soon after in the growing season by clusters of dead stems resulting from larval feeding [17,19]. Transect lines, 30 m in length, spaced every five meters, were run through the length of each stand and stems in contact with each transect line were examined for insect damage.

### 2.4. Statistical Analysis

Wilcoxon tests were used to evaluate differences in survivorship over time between native and introduced plant feeding treatments by comparing survivorship curves of *P. viator* fed native and introduced *P. australis* leaves, *R. lutosa* fed native and introduced *P. australis* rhizomes, and *R. lutosa* fed artificial diet consisting of native and introduced *P. australis* rhizomes. An intrinsic characteristic of survival data measuring time until an event, such as death, is the possibility of the event not occurring due to other factors or events (censoring). In this study, larvae that pupated or entered diapause were treated as right-censored cases in the analysis.

Insufficient *P. viator* individuals pupated to statistically evaluate differences in pupal weight and pupal duration among *P. australis* haplotype and *Z. aquatica* feeding treatments. The mean and standard deviation for these variables are provided in Table 1 for comparison. Differences in pupal weight and pupal duration between *P. viator* males and females were analyzed using a t-test. Differences in pupal weight and pupation time of *R. lutosa* larvae were analyzed by two-way ANOVA using food type (feed rhizomes or diet) and plant provenance as independent variables. A few larvae that were accidentally killed during experiments were not included in analyses.

## 3. Results

### 3.1. Poanes viator Larvae Fed Leaves

**Figure 1:**
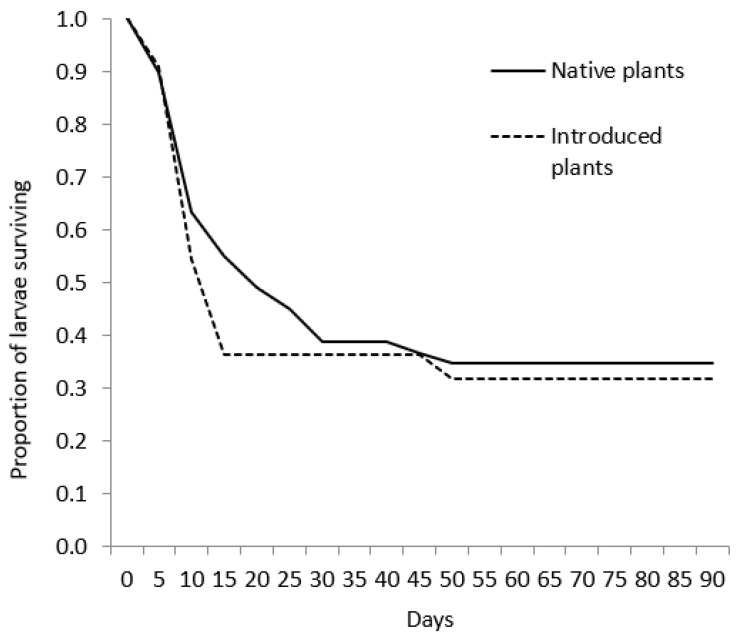
Survivorship curves for *Poanes viator* larvae fed leaves from either native plants (native *Phragmites australis* haplotypes or *Zizania aquatica*) or introduced *P. australis* haplotypes. A caterpillar was considered to have survived if it reached pupation or entered a state of diapause.

We collected 100 eggs from 45 captive *P. viator* females in July and August 2002. Of those eggs, 74 hatched and 71 larvae that survived past one day were used in treatments. There was no significant difference in survivorship curves of larvae feed native or introduced plants (W = 0.48, d.f. = 1, *p* = 0.491; Figure 1), or at the plant haplotype/species level (W = 2.36, d.f. = 5, *p* = 0.797). Larval mortality ranged from 42% and 73% among plant haplotypes/species (Table 2). Larval performance results are listed in Table 1. Larvae that died before pupation lived 11.2 ± 9.1 (±SD) days, whereas larval duration for individuals reaching pupation averaged 57.3 ± 15.2 days. Only larvae fed native *Phragmites* haplotypes or *Z. aquatica* pupated, and all of these individuals matured to adults. Some larvae from all feeding treatments entered a quiescent state (presumably diapause; Table 1). These larvae did not feed or move unless disturbed and did not pupate by the termination of this experiment. Of the larvae that pupated, average larval duration was at least 25 days longer for individuals fed haplotype AB than those fed haplotype E or *Z. aquatica*. Pupal duration averaged 13.3 ± 1.3 days and was not different among plant species or haplotypes although we note that sample sizes were low (Table 2). Pupal weight of females (0.35 ± 0.01 g, *n* = 4) was significantly greater than that of males (0.25 ± 0.01 g, *n* = 3; t = 6.03, d.f. = 5, *p* = 0.03).

### 3.2. Rhizedra lutosa Larvae Fed Rhizomes or Diet

**Figure 2:**
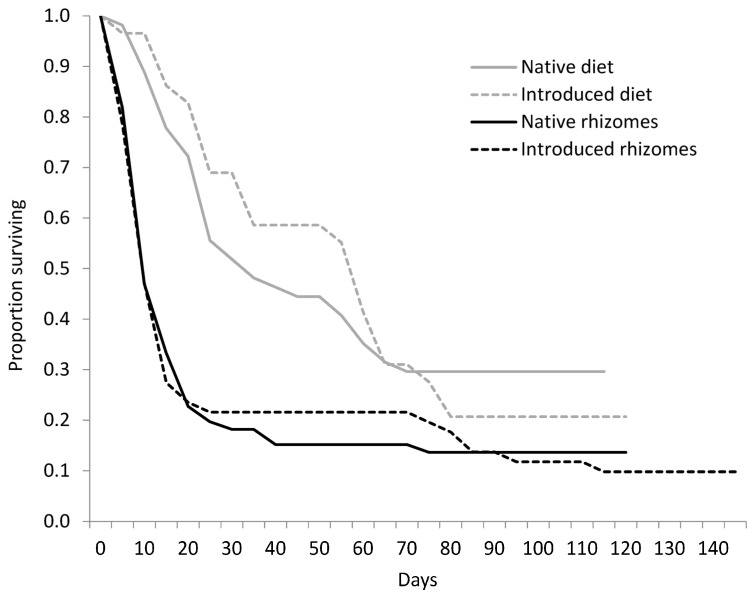
Survivorship curves for *Rhizedra lutosa* larvae under four feeding treatments. Larvae were fed either rhizome pieces from native *Phragmites australis* haplotypes, rhizome pieces from introduced *P. australis* haplotypes, diets containing ground rhizomes from native *P. australis* haplotypes, or diets containing ground rhizomes from introduced *P. australis* haplotypes.

We collected 1082 eggs from *R. lutosa* females from September to November 2003. Of those eggs, 285 hatched; 117 larvae were used in the rhizome experiment and 114 larvae were used in the diet experiment. There was no significant difference in survivorship curves between larvae fed native or introduced *P. australis* rhizomes (W = 0.04, d.f. = 1, *p* = 0.847; Figure 2), or among the plant haplotype treatments (W = 6.25, d.f. = 4, *p* = 0.181). Mortality within the first 21 days was high for all haplotypes, and larvae that died before pupation lived an average of 15.0 ± 22.2 days. Only 14 percent of larvae pupated on native haplotypes and 10 percent pupated on the introduced haplotype. For larvae that reached pupation, larval duration averaged 109.1 ± 15.8 days and was not significantly different between native and introduced haplotype treatments (Table 3). Pupation time and pupal weight did not differ between native or introduced lineages or between sexes (Table 3). Of the 12 adults that eclosed, eight were female and four were male.

**Figure 3:**
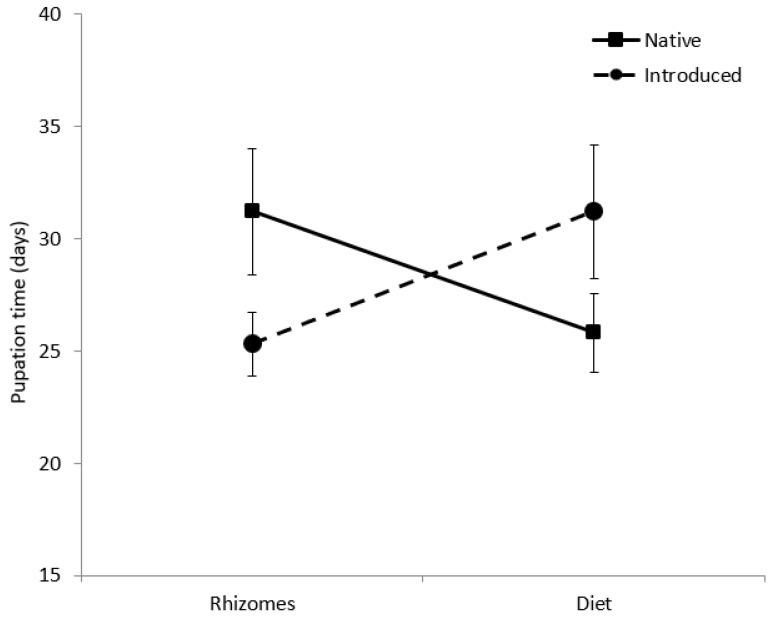
Pupation time (days) of *Rhizedra lutosa* larvae under four feeding treatments. Larvae were fed either rhizome pieces from native *Phragmites australis* haplotypes, rhizome pieces from introduced *P. australis* haplotypes, artificial diet containing ground rhizomes from native *P. australis* haplotypes, or artificial diet containing ground rhizomes from introduced *P. australis* haplotypes.

Survivorship curves of larvae fed diets were significantly different from larvae fed rhizomes (W = 18.20, d.f. = 1, *p* = 0.001; Figure 2), a trend that was constant across haplotypes. Survival to pupation was 26.5% for larvae on artificial diet, but only 12% for larvae fed rhizomes (Table 3). The diet decreased average larval development time by 28 days with the native diet treatments and by 51 days for larvae in the introduced diet treatment. Pupal weight of larvae fed diet (0.70 ± 0.17 g) was significantly greater than larvae fed rhizomes (0.54 ± 0.11 g; F_(1,26)_ = 21.7, *p* = 0.001; Table 3). Pupal weight was not affected by provenance. There was a significant interactive effect of food type and native status on pupation time (F_(1,22)_ = 5.2, *p* = 0.033; Figure 3). Larvae developed more quickly on the native diet than on the introduced diet, but the opposite trend was observed for larvae fed native and introduced rhizomes.

### 3.3. Field Surveys for Rhizedra lutosa

In 2004, we found *R. lutosa* feeding damage at low densities (average of less than one damaged stem per transect line) in only one introduced stand on Block Island. From June through September 2005, we consistently found damaged stems at low densities in both of the introduced stands on Block Island. In September 2005, we found a cluster of four damaged stems in one of the adjacent native stands on Block Island. No evidence of *R. lutosa* was found in *P. australis* stands in Maine.

## 4. Discussion

*Phragmites australis* occurs in North America as a complex of closely related native haplotypes (subspecies *americanus*) and several other introduced haplotypes from around the world [1,2,20]. Several specialist insects feed on both native and introduced lineages, but often occur in higher densities on native haplotypes, including *Hyalopterus pruni* (Hemipetera: Aphididae) [10,21] and *Lipara* spp. [22,23]. *Poanes viator* is common in wetlands through the eastern United States [13] and abundant in introduced *Phragmites* stands in southern New England (A. Lambert, personal observation). *Rhizedra lutosa* is a relatively recent introduction into the Northeast [16] that is rapidly spreading from this region, but populations generally occur at low densities and with little herbivory impacts to plants [17]. It is unclear how strongly nutritional quality differs between genetic lineages or whether the development of associated insects is affected by genetic origin (but see [11]).

Overall, survival to pupation was relatively low for both insects with no detectable difference in longevity or survival rate based on host plant species or *P. australis* lineage. Most mortality occurred early after hatching, and this is a common but difficult to explain phenomenon in Lepidoptera [24]. In *Helicoverpa armigera* (Lepidoptera: Noctuidae) feeding on cotton, mortality of first instar larvae can be as high as 93–100% [25]. Life table studies have provided limited understanding of the factors that contribute to larval mortality but most often neonate mortality is attributed to unknown factors [25,26]. Host plant quality can have strong but variable effects on larval survival and development [27,28] often associated with plant secondary chemistry [28,29]. In the milkweed specialist *Danaus plexippus* (Lepidoptera: Nymphalidae), mortality was highest in first instar larvae (ranging from 39 to 100% depending on plant species) and was dependent on leaf tissue quality, especially defensive chemical concentrations [24]. Although we did not directly test differences in host plant quality (nutrients, defensive chemistry, etc.) among plant species or lineages, this is an important consideration when evaluating herbivore performance, especially as part of a biological control program where high survival is an important quality for successful implementation.

*Poanes viator* is an oligophagous herbivore that feeds on a variety of monocots [13], and in our study, larvae performed similarly on all plants tested. Although caterpillars from all treatments reached diapause or pupation, only individuals feeding on native plants developed to adults. Due to the low final sample sizes, we cannot definitively say whether native plants are higher quality hosts. Balme [19] successfully reared *P. viator* larvae collected from an introduced *P. australis* stand to adults using introduced leaves, but had no success rearing insects starting from eggs. In surveys of introduced *Phragmites* stands throughout Rhode Island, we routinely find *P. viator* pupae and adults in most stands and with no other host plants available (A. Lambert, unpublished data), providing evidence that butterflies do complete their life cycle on this lineage. Two possible mechanisms may account for the increased use of introduced *P. australis* observed in wild *P. viator* populations: (1) invasion of introduced *P. australis* into wetlands reduces or excludes the normal food plants of *P. viator* forcing the butterflies to feed on the introduced haplotype, and/or (2) *P. viator* is opportunistic and is incorporating this increasingly abundant plant into its diet. As introduced *P. australis* has invaded across the range of *P. viator*, native plants have become less abundant in wetland habitats [3,30], including native *P. australis* [2]. The rapid expansion of introduced *P. australis* over the past century [24] may have necessitated a shift in host plants by *P. viator* [31], as well as facilitated the recent increase in its range [13,14].

We expected that *R. lutosa*, a European insect, would only survive on introduced *P. australis* (of European ancestry) since it has only been reported to occur in this haplotype in North America [16,17]. However, larvae were able to complete development on all haplotypes tested. While working in *P. australis* stands over several years and in the surveys reported here, we commonly encountered larval damage and collected adult moths in introduced *P. australis* stands throughout southern New England, but only found one occurrence of larval use of native *P. australis* on Block Island. We do not know if this was incidental feeding or perhaps spillover from the adjacent introduced stand, or if the insects are maintaining populations on native plants. Only one individual fed rhizomes from this Block Island population survived to adult and this individual also had the lowest pupal weight and longest pupation time in this study. As *R. lutosa* expands south and west, it will continue to encounter other native *P. australis* haplotypes.

Although *R. lutosa* survival to pupation was low on all haplotypes tested, eclosion rates were high. There are several issues that may have contributed to low survival. Under natural conditions, larvae most likely feed continuously within the network of rhizomes. In our study, every few days, larvae needed to be removed from each rhizome and introduced into a new rhizome fragment by hand. Larvae did not always stay inside and were sometimes found wandering in containers, so these individuals may not have received adequate nutrition. Further, native rhizomes are generally narrower in diameter than introduced rhizomes (unpublished data), and we had difficulty supplying native rhizomes of adequate size to support feeding of late instars. In these cases, rhizomes were split longitudinally and the halves were provided to large larvae. We assume that inability to continuously feed internally on rhizomes and lack of appropriate size rhizomes adversely affected larvae and possibly increased mortality rates. Introduced plants also generally produced new rhizomes quicker, and we assume these younger rhizomes were of greater nutritional quality than older ones that were used for part of the native feeding treatments when new rhizomes were not available. Further, pupal weight was consistently higher and pupal time shorter for larvae fed introduced rhizomes. The difference observed in pupal weight between native and introduced plants was partly skewed by the one individual that survived on haplotype AB rhizomes (Table 3). This native haplotype from Block Island was difficult to grow and produced low quality rhizomes, and most larvae did not appear to do well on it.

The diet was an acceptable alternative to rhizomes as larval survival and development was higher on diet than on rhizomes, regardless of haplotype. Growing and collecting sufficient amounts of rhizomes was difficult and often the limiting factor in our ability to run replicates. The diet required only a fraction of the rhizomes that were needed to rear insects on rhizomes alone, and could be made in advance and stored, reducing the labor needed to rear insects. This diet may also be suitable for other Lepidopteran larvae that being considered as biocontrol agents against invasive *P. australis* [6,7,32]. 

This is the first study we know of comparing herbivore performance on a native host haplotype and an introduced haplotype. Our results and field studies indicate that at least for these insect species, nutritional quality of native and introduced lineages is adequate for completing development. Several laboratory studies have shown that insect herbivores will readily feed on novel invasive plant genotypes when experimentally confined on plants [33,34,35], but may not do so under natural conditions [34]. Thus, studies are needed to determine the effects of *P. australis* haplotype/lineage on insect development under field conditions. Plant traits that can vary by genotype, such as secondary chemistry, structural defenses, nutritional quality, and architecture, can differentially affect the performance of insect herbivores [36,37,38]. We expect that physical or chemical differences between native and introduced *P. australis* would prevent use by highly specialized insects that do not have an evolutionary history with a particular lineage. There is evidence of this occurring in North America. For example, although some insects commonly occur on both lineages [21,22,23], three native herbivores, *Calamomyia phragmites* (Diptera: Cecidomyiidae), *Thrypticus willestoni* (Hymenoptera: Dolichopodidae), and *Ochlodes Yuma* (Lepidopter: Hesperiidae), have been found exclusively on the native lineage and four introduced specialists, *Lasioptera hungarica* (Diptera: Cecidomyiidae), *Giraudiella inclusa* (Diptera: Cecidomyiidae), *Lipara pullitarsus* (Diptera: Chloropidae) and *R. lutosa*, have only been recorded on introduced *P. australis* [5].

Plant species and genotype can strongly influence the fecundity of insect herbivores [39], as well as potentially reduce the effectiveness of biological control agents [35,37,40]. Isolating the factors that influence plant quality may help clarify the mechanisms behind the different growth responses observed in *P. viator* and *R. lutosa* feeding on different plant haplotypes. These considerations are vital to our understanding of plant-insect interactions, especially in light of ongoing efforts to find haplotype-specific agents for the control of invasive *P. australis*. As more European insects are tested, we will gain a clearer picture of the potential of finding a haplotype specific biological control agent for the management of introduced *P. australis*.

## Figures and Tables

**Table 1:** Development results for feeding experiments where *Poanes viator* larvae were fed leaves from native *Phragmites australis* subsp. *americanus* haplotypes, introduced *P. australis* haplotypes, or *Zizania aquatica*. Replicates with larvae entering diapause were terminated after 93 days and are not included. Means ± standard deviations are given.

Plant Species	Native Status	N	Larval Duration of Individuals Not Reaching Pupation (Days ± SD)	Larval Duration of Individuals Reaching Pupation (Days ± SD)	N Pupated	Pupal Weight (g)	Pupal Duration (Days)	N Eclosed
*Phragmites australis*								
Haplotype M (RI)	Introduced	11	13.6 ± 14.6	-	0	-	-	-
Haplotype M (NY)	Introduced	11	8.3 ± 3.9	-	0	-	-	-
Haplotype E (ME)	Native	11	8.6 ± 2.2	-	0	-	-	-
Haplotype E (NY)	Native	12	8.4 ± 8.1	49.6 ± 11.8	3	0.26 ± 0.01	13.0 ± 1.4	2
Haplotype AB (RI)	Native	13	10.3 ± 9.2	78.5 ± 6.4	2	0.34 ± 0.04	12.0 ± 1.4	2
*Zizania aquatica*	Native	13	16.6 ± 10.3	52.0 ± 1.0	3	0.32 ± 0.07	13.7 ± 1.5	3

**Table 2:** Survival of *Poanes viator* larvae to pupation and adulthood. Larvae were fed leaves from native *Phragmites australis* subsp. *americanus* haplotypes, introduced *P. australis* haplotypes, or *Zizania aquatica*. Means ± standard deviations are given.

Plant Species	Initial N	N Died in Larval Stage	N Entering Diapause	N Pupated	N Eclosed
*Phragmites australis*					
Haplotype M (RI)	11	7	4	0	-
Haplotype M (NY)	11	8	3	0	-
Haplotype E (ME)	11	7	4	0	-
Haplotype E (NY)	12	5	5	2	2
Haplotype AB (RI)	13	9	2	2	2
*Zizania aquatica*	13	9	1	3	3
Native plants	49	30	12	7	7
Introduced plants	22	15	7	0	0

**Table 3:** Development results for feeding experiments where *Rhizedra lutosa* larvae were fed rhizomes from either native *Phragmites australis* subsp. *americanus* haplotypes, introduced *P. australis* haplotypes, or *Zizania aquatica*. Results are also given for a feeding experiment where *Rhizedra lutosa* larvae were fed different artificial diets with either native or introduced *P. australis* rhizomes.

Haplotype	Native Status	N	Larval Duration of Individuals Not Reaching Pupation (Days ± SD)	Larval Duration of Individuals Reaching Pupation (Days ± SD)	N Pupated	Pupal Weight (g)	Pupal Duration (days)	N Eclosed
*R. lutosa* fed rhizomes								
Haplotype M (RI)	Introduced	25	24.0 ± 37.1	122.0 ± 16.3	4	0.47 ± 0.03	24.0± 2.8	3
Haplotype M (NY)	Introduced	27	15.1 ± 22.9	82	1	no data	25	1
Haplotype E (ME)	Native	32	9.2 ± 7.7	106.3 ± 11.0	3	0.62 ± 0.07	27.3 ± 2.1	3
Haplotype E (NY)	Native	22	13.0 ± 8.6	100.0 ± 8.8	4	0.46 ± 0.08	29.5 ± 5.7	4
Haplotype AB (RI)	Native	11	33.7 ± 46.5	119.0 ± 7.1	2	0.29 ± 0.08	38	1
*Native haplotypes*		66	11.3 ± 11.3	106.3 ± 11.2	9	0.48 ± 0.15	29.6 ± 5.4	8
*Introduced haplotype*		51	19.3 ± 30.0	114.0 ± 22.8	5	0.52 ± 0.07	25.3 ± 2.5	4
*R. lutosa* fed diet								
Haplotype M (RI)	Introduced	28	48.4 ± 36.8	66.2 ± 13.7	6	0.74 ± 0.11	31.2 ± 6.7	5
Haplotype E (NY)	Native	27	31.7 ± 24.2	80.4 ± 19.8	9	0.64 ± 0.16	24.6 ± 5.4	8
Haplotype AB (RI)	Native	28	33.1 ± 527.2	73.8 ± 25.0	7	0.73 ± 0.21	27.6 ± 4.5	5

## Data Availability

All data presented in this study are contained within the article.

## References

[1] Lambert A.M., Saltonstall K., Long R., Dudley T.L. (2016). Biogeography of *Phragmites australis* lineages in the southwestern United States. Biol. Invasions.

[2] Saltonstall K. (2002). Cryptic invasion by a non-native genotype of the common reed, *Phragmites australis*, into North America. Proc. Natl. Acad. Sci. USA.

[3] Chambers R.M., Meyerson L.A., Saltonstall K. (1999). Expansion of *Phragmites australis* into tidal wetlands of North America. Aquat. Bot..

[4] Meyerson L.A., Lambert A.M., Saltonstall K. (2010). A tale of three lineages: Expansion of common reed (*Phragmites australis*) in the U.S. Southwest and gulf coast. Invasive Plant Sci. Manag..

[5] Blossey B., Casagrande R.A. (2016). Biological control of invasive *Phragmites* may safeguard native *Phragmites* and increase wetland conservation values. Biol. Invasions.

[6] Blossey B., Häfliger P., Tewksbury L., Dávalos A., Casagrande R.A. (2018). Host specificity and risk assessment of *Archanara geminipuncta* and Archanara neurica, two potential biocontrol agents for invasive *Phragmites australis* in North America. Biol. Control.

[7] Tewksbury L., Casagrande R.A., Blossey B., Hafliger P., Schwarzlander M. (2002). Potential for biological control of *Phragmites australis* in North America. Biol. Control.

[8] Blossey B., Endriss S.B., Casagrande R., Häfliger P., Hinz H., Dávalos A., Brown-Lima C., Tewksbury L., Bourchier R.S. (2020). When misconceptions impede best practices: Evidence supports biological control of invasive Phragmites. Biol. Invasions.

[9] Cronin J.T., Kiviat E., Meyerson L.A., Bhattarai G.P., Allen W.J. (2016). Biological control of invasive *Phragmites australis* will be detrimental to native *P. australis*. Biol. Invasions.

[10] Bhattarai G.P., Meyerson L.A., Cronin J.T. (2017). Geographic variation in apparent competition between native and invasive *Phragmites australis*. Ecology.

[11] Cronin J.T., Bhattarai G.P., Allen W.J., Meyerson L.A. (2015). Biogeography of a plant invasion: Plant–herbivore interactions. Ecology.

[12] Kendall R.O. (1966). Larval food plants and distribution notes for three Texas Hesperiidae. J. Lepid. Soc..

[13] Shapiro A.M. (1970). Notes on the biology of *Poanes viator* (Hesperiidae) with the description of a new subspecies. J. Res. Lepid..

[14] Gochfeld M., Burger J. (1997). Butterflies of New Jersey.

[15] van der Toorn J., Mook J.H. (1982). The influence of environmental factors and management on stands of *Phragmites australis.* I. Effects of burning, frost and insect damage on shoot density and shoot size. J. Appl. Ecol..

[16] McCabe T.L., Schweitzer D.F. (1991). *Rhizedra lutosa* (Lepidoptera: Noctuidae) newly introduced to North America. Entomol. News.

[17] Casagrande R.A., Balme G., Blossey B. (2003). *Rhizedra lutosa*, a natural enemy of *Phragmites australis* in North America. Estuaries.

[18] Mikkola K., Lafontaine J.D. (1994). Recent introductions of riparian noctuid moths from the Palearctic region to North America, with the first report of *Apamea unanimis* (Huebner) (Noctuidae; Amphipyrinae). J. Lepid. Soc..

[19] Balme G. (2000). Insects on *Phragmites australis*. Master’s Thesis.

[20] Lambertini C., Mendelssohn I.A., Gustafsson M.H.G., Olesen B., RIIS T., Sorrell B.K., Brix H. (2012). Tracing the origin of Gulf Coast *Phragmites* (Poaceae): A story of long-distance dispersal and hybridization. Am. J. Bot..

[21] Lambert A.M., Casagrande R.A. (2007). Susceptibility of native and non-native common reed to the non-native mealy plum aphid (Homoptera: Aphididae) in North America. Environ. Entomol..

[22] Allen W.J., Young R.E., Bhattarai G.P., Croy J.R., Lambert A.M., Meyerson L.A., Cronin J.T. (2015). Multitrophic enemy escape of invasive *Phragmites australis* and its introduced herbivores in North America. Biol. Invasions.

[23] Lambert A.M., Winiarski K., Casagrande R.A. (2007). Distribution and impact of exotic gall flies (*Lipara* sp.) on native and exotic *Phragmites australis*. Aquat. Bot..

[24] Zalucki M.P., Clarke A.R., Malcolm S.B. (2002). Ecology and behavior of first instar larval lepidoptera. Annu. Rev. Entomol..

[25] Kyi A., Zalucki M.P., Titmarsh I.J. (1991). An experimental study of early stage survival of *Helicoverpa armigera* (Lepidoptera: Noctuidae) on cotton. Bull. Entomol. Res..

[26] Titmarsh I.J. (1992). Mortality of Immature Lepidoptera: A Case Study with *Heliothis* Species (Lepidoptera: Noctuidae) in Agricultural Crops on the Darling Downs. Ph.D. Thesis.

[27] Barnes E.E., Gosnell S., Hallagan C., Otten K.E., Slayter L., Murphy S.M. (2016). Performance of Western tent caterpillar (*Malacosoma californicum*) on two common host plants, including a new host plant record. J. Lepid. Soc..

[28] Singer M.S., Rodrigues D., Stireman J.O., Carrière Y. (2004). Roles of food quality and enemy-free space in host use by a generalist insect herbivore. Ecology.

[29] Berenbaum M.R., Zangerl A.R. (1998). Chemical phenotype matching between a plant and its insect herbivore. Proc. Natl. Acad. Sci. USA.

[30] Meyerson L.A., Saltonstall K., Windham L., Kiviat E., Findlay S. (2000). A comparison of Phragmites australisin freshwater and brackish marsh environments in North America. Wetl. Ecol. Manag..

[31] Nakamura I., Cooper D.R. (2005). An inland population of *Poanes viator* (Hesperiidae) associated with *Phragmites australis*, the common reed. J. Lepid. Soc..

[32] Häfliger P., Teyssiere S. (2004). Evaluating the Potential for Biological Control of Common Reed, Phragmites Australis.

[33] Bossdorf O., Schroder S., Prati D., Auge H. (2004). Palatability and tolerance to simulated herbivory in native and introduced populations of Alliaria petiolata (Brassicaceae). Am. J. Bot..

[34] Siemann E., Rogers W.E. (2003). Reduced resistance of invasive varieties of the alien tree *Sapium sebiferum* to a generalist herbivore. Oecologia.

[35] Wang Y., Huang W., Siemann E., Zou J., Wheeler G.S., Carrillo J., Ding J. (2011). Lower resistance and higher tolerance of invasive host plants: Biocontrol agents reach high densities but exert weak control. Ecol. Appl..

[36] Levin D.A. (1973). The Role of trichomes in plant defense. Q. Rev. Biol..

[37] Manrique V., Cuda J.P., Overholt W.A., Williams D.A., Wheeler G.S. (2008). Effect of host-plant genotypes on the performance of three candidate biological control agents of *Schinus terebinthifolius* in Florida. Biol. Control.

[38] Underwood N., Rausher M.D. (2000). The effects of host-plant genotype on herbovire population dynamics. Ecology.

[39] Awmack C.S., Leather S.R. (2002). Host plant quality and fecundity in herbivorous insects. Annu. Rev. Entomol..

[40] Garcia-Rossi D., Rank N., Strong D.R. (2003). Potential for self-defeating biological control? Variation in herbivore vulnerability among invasive *Spartina* genotypes. Ecol. Appl..

